# Adaptive deep brain stimulation for Parkinson's disease demonstrates reduced speech side effects compared to conventional stimulation in the acute setting

**DOI:** 10.1136/jnnp-2016-313518

**Published:** 2016-08-16

**Authors:** Simon Little, Elina Tripoliti, Martijn Beudel, Alek Pogosyan, Hayriye Cagnan, Damian Herz, Sven Bestmann, Tipu Aziz, Binith Cheeran, Ludvic Zrinzo, Marwan Hariz, Jonathan Hyam, Patricia Limousin, Tom Foltynie, Peter Brown

**Affiliations:** 1 Sobell Department of Motor Neuroscience & Movement Disorders, UCL Institute of Neurology, London, UK; 2 Department of Neurology, University Medical Centre Groningen, University of Groningen, Groningen, The Netherlands; 3 MRC Brain Network Dynamics Unit (BNDU), Department of Pharmacology and Nuffield Department of Clinical Neurosciences, University of Oxford, Oxford, UK; 4 Nuffield Department of Surgical Sciences, John Radcliffe Hospital, University of Oxford, Oxford, UK

**Keywords:** PARKINSON'S DISEASE, SPEECH

## Introduction

Deep brain stimulation (DBS) for Parkinson's disease (PD) is currently limited by costs, partial efficacy and surgical and stimulation-related side effects. This has motivated the development of adaptive DBS (aDBS) whereby stimulation is automatically adjusted according to a neurophysiological biomarker of clinical state, such as β oscillatory activity (12–30 Hz). aDBS has been studied in parkinsonian primates and patients and has been reported to be more energy efficient and effective in alleviating motor symptoms than conventional DBS (cDBS) at matched amplitudes.^1^ ^2^

However, these studies have not considered whether side effects can also be avoided with clinically effective stimulation. In PD, it is well recognised that a significant proportion of patients develop speech deterioration following DBS of the subthalamic nucleus (STN), which may be reversible.^3^

Here we test bilateral stimulation, optimising parameters for aDBS, and evaluate speech intelligibility. We hypothesised that acute aDBS would be more effective and more efficient than cDBS at matched stimulation parameters while causing less speech impairment.

## Methods

We recruited 10 patients with advanced idiopathic PD following implantation of DBS electrodes into the STN.^2^ Recordings took place 3–6 days following electrode placement during a temporary period of externalisation. All participants gave informed written consent, and were tested following overnight withdrawal of dopaminergic medication (see online supplementary material). Two patients were excluded due to external stimulator failure leading to no voltage delivery under aDBS and cDBS conditions.

10.1136/jnnp-2016-313518.supp1 supplementary data

aDBS stimulation was delivered bilaterally, only when β amplitude exceeded a threshold as previously described.^2^ aDBS contacts, voltages and trigger thresholds were independently set for the two sides according to motor benefit versus induced paraesthesiae, with the same contacts/voltages used for cDBS.

Stimulation in each block continued for 15 min prior to evaluation. Participants were assessed during blinded and randomised aDBS, cDBS and OFF conditions using the standardised and validated speech intelligibility test (SIT) in which participants read sentences totalling 110 words.^4^ ^5^ Speech was recorded, and % intelligibility was assessed by a speech and language therapist (blinded to condition). Six of the eight participants completed an MDS-UPDRS-III assessment, which was videoed and rated off-line (rigidity excluded, vUPDRS total=112) by two blinded movement disorder specialists. Two participants did not perform the MDS-UPDRS-III assessment due to fatigue/discomfort related to prolonged off states. Our primary outcome measures were a comparison of aDBS with cDBS for speech (SIT), and for motor impairment (vUPDRS). Statistical testing was performed by repeated measures ANOVA (rmANOVA) and the Student's t-test.

## Results

The mean voltage (fixed across cDBS and aDBS) was 2.7±0.2 V, with stimulation in the aDBS condition delivered 42.6±3.7% of the time.

### Speech scores

**Figure 1:**
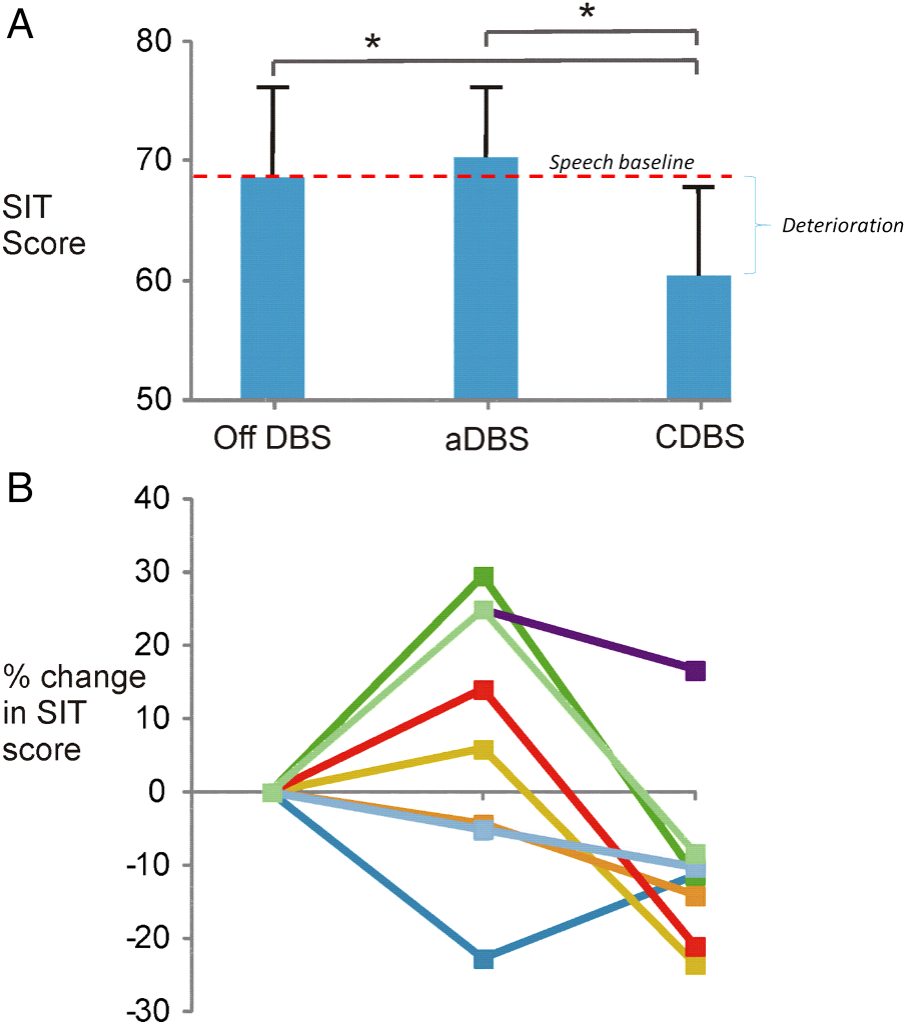
(A) Bar chart showing the mean±SEM speech intelligibility across the three stimulation conditions for all eight patients. (B) Data show individual percentage change for all participants across stimulation conditions, normalised to the Off DBS state. aDBS, adaptive DBS; cDBS, conventional DBS; DBS, deep brain stimulation; SIT, speech intelligibility test.

Baseline SIT scores OFF medication were 67.9±9.2%. rmANOVA (Off DBS, aDBS and cDBS) demonstrated a significant main effect of stimulation type (F_2_=4.153, p=0.038). Our planned contrast demonstrated better speech intelligibility with aDBS (70.4±6.4%) than with cDBS (60.5±8.2%; t_7_=2.8, p_two tailed_=0.02; figure 1). In secondary exploratory comparisons, aDBS was no different to off DBS, but cDBS was worse than off DBS (t_7_=2.55, p_two tailed_=0.038).

### Motor scores

Baseline vUPDRS-III scores, OFF medication, were 28.8±4.5 (6 participants). This was compared to a mean preoperative score of 36.4, suggestive of a microlesional effect of surgery. The vUPDRS-III score across the three conditions (Off DBS, aDBS and cDBS) was compared by rmANOVA (6 participants) and demonstrated a significant main effect of stimulation (F_df=2_=5.4, p=0.025). Our planned contrast revealed a significant improvement of aDBS compared to cDBS (vUPDRS-III means: 19.7±1.0 vs 31.6±4.3; t_5_=2.71, p_two tailed_=0.042).

## Discussion

Recent work has demonstrated that aDBS may be more effective at improving motor symptoms than conventional stimulation in PD with stimulation amplitudes optimised for cDBS.^1^ ^2^ Here, we investigate acute stimulation-induced speech deterioration with parameters optimised for aDBS contrasted with cDBS using these same parameters. We found that in this acute setting, the stimulation parameters optimal for aDBS significantly reduced reversible speech side effects and improved motor function, whereas these same stimulation parameters failed to produce a beneficial effect with cDBS and led to impairment in speech intelligibility. Together these findings suggest that stimulation parameters adapted to aDBS may potentially have a wider therapeutic window than cDBS with the same parameters. This dual effect whereby aDBS appears to have a lower efficacy threshold but spares speech may be related to the temporal targeting of β bursts by stimulation.

The average deterioration in the SIT score when stimulated by cDBS as opposed to aDBS was clinically relevant (9.9%). For quantitative comparison, the average deterioration when the STN is stimulated at 4 V compared to 2 V is reported as 16.5%,^4^ and the average improvement with Lee Silverman Voice Treatment was most recently reported as 4.7%.^5^

The present study has some acknowledged limitations mostly stemming from its acute nature in the postoperative period resulting in a temporary microlesional effect. Consequently, the responses to stimulation may not necessarily be representative of the chronic state. Furthermore, the postoperative period also introduced time constraints and patients were significantly fatigued by testing, and only short stimulation blocks were performed that may have lessened the mean effect of stimulation and increased the variability. In addition, our sample size was limited, and the use of blinded video-based assessments, which necessitates excluding rigidity scores and provides smaller effect sizes than non-blinded observations, may have obscured beneficial effects of cDBS. In sum, the current study cannot confirm whether aDBS will prove more effective or tolerable than independently optimised cDBS in the chronic setting but does provide proof of concept data that aDBS may, at least acutely, have less propensity for causing unwanted side effects than cDBS.

In conclusion, our study provides the first blinded group data demonstrating that aDBS has the potential to be more efficacious, with lower stimulation efficacy thresholds and less speech side effects than cDBS, although this will need confirmation in trials in chronically implanted patients.

## References

[1] Rosin B, Slovik M, Mitelman R, et al Closed-loop deep brain stimulation is superior in ameliorating parkinsonism. Neuron 2011;72:370–84. 10.1016/j.neuron.2011.08.023 22017994

[2] Little S, Pogosyan A, Neal S, et al Adaptive deep brain stimulation in advanced Parkinson disease. Ann Neurol 2013;74:449–57. 10.1002/ana.23951 23852650 PMC3886292

[3] Skodda S, Grönheit W, Schlegel U, et al Effect of subthalamic stimulation on voice and speech in Parkinson's disease: for the better or worse? Front Neurol 2014;4:218 10.3389/fneur.2013.00218 24454305 PMC3888994

[4] Tripoliti E, Zrinzo L, Martinez-Torres I, et al Effects of contact location and voltage amplitude on speech and movement in bilateral subthalamic nucleus deep brain stimulation. Mov Disord 2008;23:2377–83. 10.1002/mds.22296 18785648

[5] Cannito MP, Suiter DM, Beverly D, et al Sentence intelligibility before and after voice treatment in speakers with idiopathic Parkinson's disease. J Voice 2012;26:214–19. 10.1016/j.jvoice.2011.08.014 22209057

