# Supplementary material for: Adaptive deep brain stimulation for Parkinson's disease demonstrates reduced speech side effects compared to conventional stimulation in the acute setting
Source: J Neurol Neurosurg Psychiatry. 2016 Aug 16;87(12):1388–9. doi: 10.1136/jnnp-2016-313518 (PMC5136720; doi:10.1136/jnnp-2016-313518)
Supplement: supplementary data [file jnnp-2016-313518supp.pdf]

				PRE – OPERATIVE UPDRS-III		Stimulation Parameters (Voltage & Contact)			
**Patient**	**Age**	**Disease duration**	**Site**	**OFF Levodopa**	**ON Levodopa**	**L**	**R**	**DBS indication**	**LED**
**1**	53	8	UCL	41	11	2.2V, 2	2.2V,1	Right tremor	560
**2**	57	4	UCL	47	4	2.8V,2	2.7V,2	Motor fluctuations/ dystonia	2040
**3**	40	4	UCL	50	21	3.5V,1	3.6V,1	Motor fluctuations	1220
**4**	57	8	UCL	42	29	2.8V,1	2.7V,1	Motor fluctuations / dyskinesias	1500
**5**	50	4	UCL	37	17	2.6V,2	2.8V,2	Motor fluctuations	750
**6**	67	6	OX	46	21	3.3V,1	3.3V,1	Tremor	1115
7	62	14	OX	58	18	2.1V,0	2.1V,0	Motor fluctuations	960
8	53	8	UCL	- *	- *	1.9V,0	1.9V,0	Motor fluctuations / dyskinesia	1300
**Mean**	**54**	**7**		**45.8**	**17.3**	**2.7**	**2.7**		**1180**
**SEM**	**2.9**	**1.2**		**2.6**	**3.0**	**0.2**	**0.2**		**162.6**

Table 1. Patient pre-operative clinical characteristics including pre-op levodopa testing using original UPDRS-III score system (total 108). Note patients shown in bold also completed additional motor UPDRS-III assessments during our study. LED = Levodopa equivalent dose. ** Patient had Levodopa challenge with > 50% response however this was 4 years prior to study date and not repeated pre-operatively at patient's request.*
